# Supplementary material for: CAR-neutrophil mediated delivery of tumor-microenvironment responsive nanodrugs for glioblastoma chemo-immunotherapy
Source: Nat Commun. 2023 Apr 20;14:2266. doi: 10.1038/s41467-023-37872-4 (PMC10119091; doi:10.1038/s41467-023-37872-4)

Supplementary Information

This file includes:

Supplementary Figures 1-13

Supplementary Tables 1-2

Source Data-Supplementary Figure 1e, 5a & 5c

Supplementary Figures

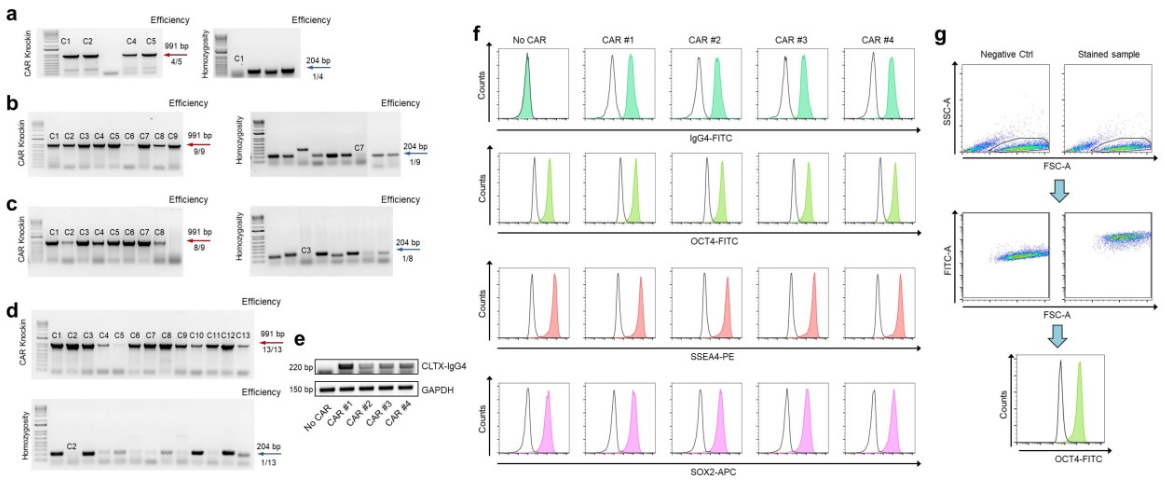

Supplementary Figure 1. **Genotyping of CAR-engineered hPSCs**. Genotyping of single cell-derived hPSC clones after puromycin selection for CAR #1 (a), CAR #2 (b), CAR #3 (c), and CAR #4 (d) is shown. Expected PCR product for correctly targeted *AAVS1* site is 991 bp (red arrow), and homozygosity assay was performed on the knock-in clones, and those without ~240 bp PCR products were homozygous (blue arrow). (e-g) RT-PCR (e) and flow cytometry (f) analysis of CLTX-IgG4 CAR and pluripotent marker expression on wildtype and CAR knock-in hPSCs are shown. (g) FACS sequential gating strategies for FACS experiments in Supplementary Figure 1f, Supplementary Figure 2, Supplementary Figure 7a, Figure 3g. For a-e, triplicates were performed independently.

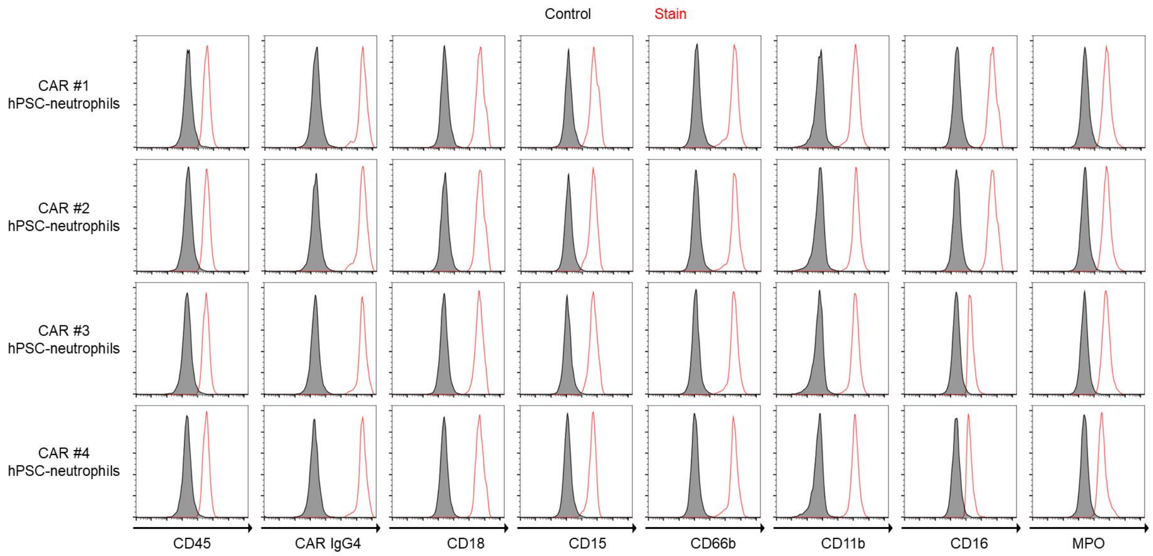

Supplementary Figure 2. **Flow cytometry analysis of CAR-neutrophils derived from various hPSCs**. Plots show unstained control (black) and specific antibody (red) histograms. These results were representative of three biologically independent experiments.

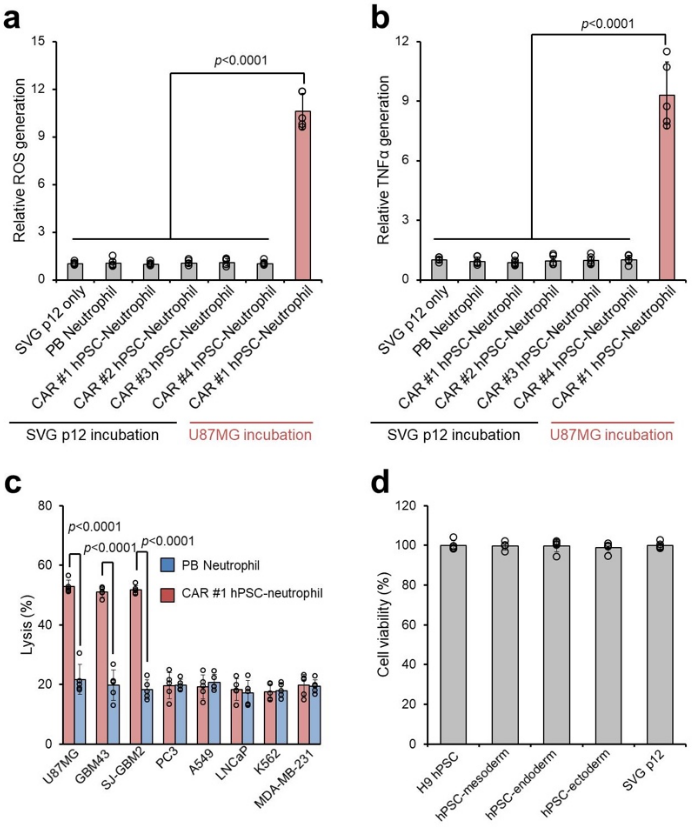

Supplementary Figure 3. Specificity and biocompatibility analysis of CAR-neutrophils. (a-b) Relative reactive oxygen species (ROS) generation (a) and TNFα release (b) from indicated neutrophil groups after co-culture with SVG p12 or U87MG cells were measured. Data are represented as mean ± SD of five independent biological replicates, one-way analysis of variance (ANOVA). (c) Cytotoxicity ability of peripheral blood (PB) or CAR neutrophils against indicated tumor cells at an effector-to-target ratio of 3: 1 are shown. For glioblastoma cell killing, U87MG cell line, primary adult GBM43, and pediatric SJ-GBM2 cells were employed. Data are represented as mean ± SD of five independent replicates, two-tailed Student's *t*-test. (d) CAR neutrophils were incubated with normal SVG p12 glial cells, H9 hPSCs, hPSC-derived mesoderm, endoderm and ectoderm at a neutrophil-to-target ratio of 10:1. The numbers of viable cells were quantified. Data are represented as mean ± SD of five independent biological replicates. Source data are provided in the Source data files.

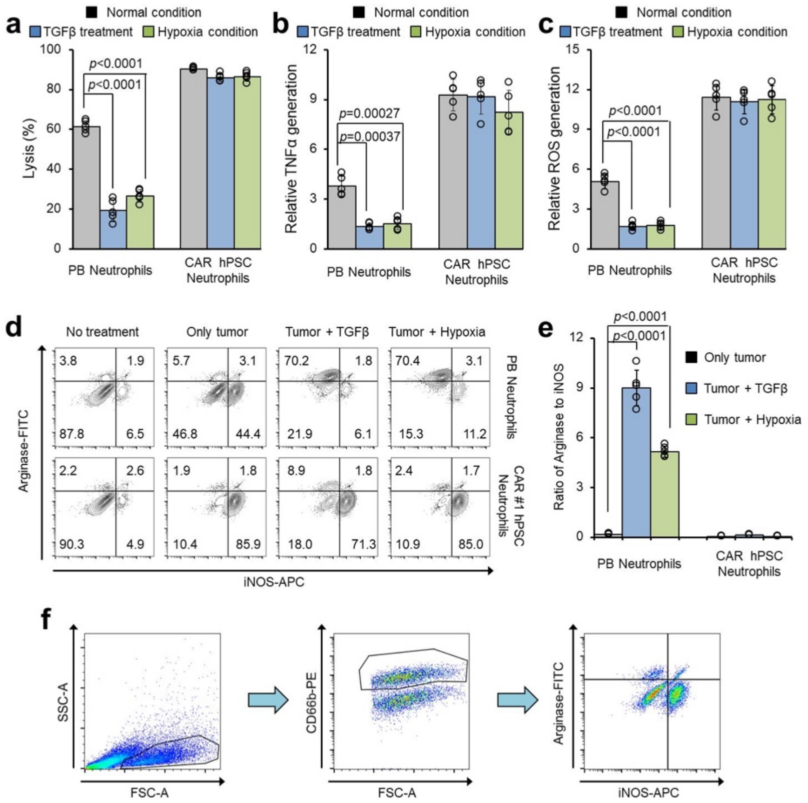

Supplementary Figure 4. CAR neutrophils, but not primary neutrophils, sustained superior antitumor activities under immunosuppressive tumor microenvironment. (a) Cytotoxicity assays against U87MG glioblastoma cells were performed at different ratios of neutrophil-to-tumor target using CAR-neutrophils or peripheral blood (PB) neutrophils under indicated conditions, n=5 biologically independent samples. (b-c) Reactive oxygen species (ROS) generation (b) and ELISA analysis of TNFα release (c) from different CAR- or PB-neutrophils after coculturing with U87MG cells were determined under indicated conditions, n=5 biologically independent samples. (d-f) Flow cytometry analysis of arginase and iNOS expression on CAR- or PB-neutrophils under indicated conditions was shown in (d) and quantified in (e), n=5 biologically independent samples. (f) FACS sequential gating strategies for FACS experiments in Supplementary Figure 4d, Supplementary Figure 7b, and Supplementary Figure 9e. All data in this figure are represented as mean ± SD, two-tailed Student's *t*-test, $*p<0.05$. Source data are provided in the Source data files.

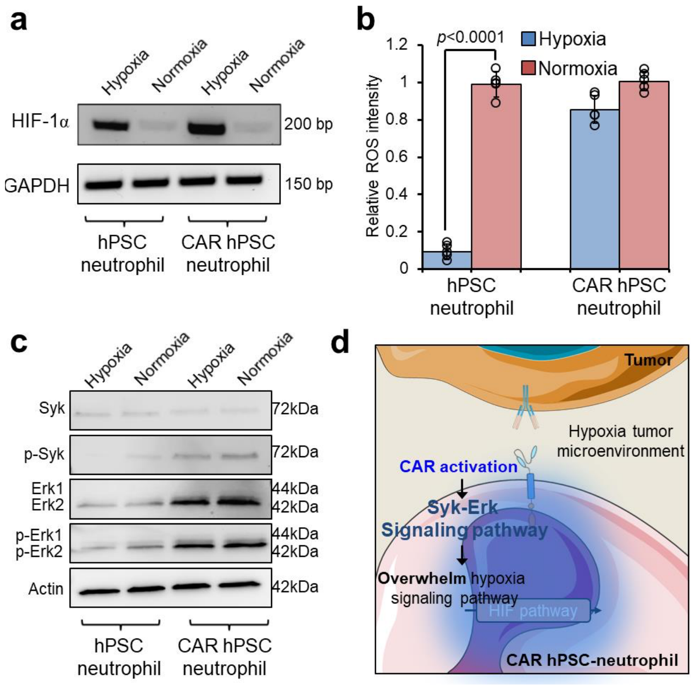

Supplementary Figure 5. Intracellular CAR signaling activation overwhelmed the influence of hypoxic tumor microenvironment. (a) RT-PCR analysis of HIF-1α expression in hPSC-neutrophils and CAR hPSC-neutrophils under normoxia or hypoxia. (b) Reactive oxygen species (ROS) generation during the disruption of tumor cells by hPSC-neutrophils and CAR hPSC-neutrophils under normoxia or hypoxia. Data are represented as mean ± SD of five independent biological replicates. Two-tailed Student's *t*-test, *$p<0.05$. Source data are provided in the Source data files. (c) Total and phosphor-protein analysis of the Syk-Erk signaling pathway in cell lysates of neutrophils via western blot was performed with co-incubation of tumor cells under normoxia and hypoxia. (d) Schematic of potential molecular mechanism underlying cancer stimulated intracellular CAR signaling activation overwhelmed the influence of hypoxic tumor microenvironment. For a and c, triplicates were performed independently.

.

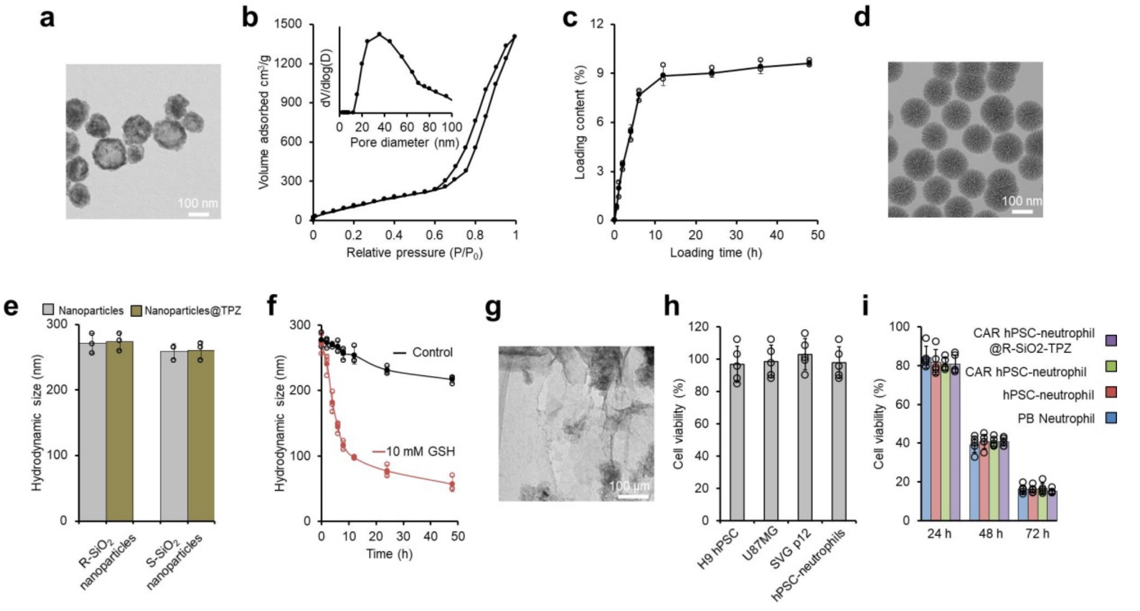

Supplementary Figure 6. Properties of smooth (S-SiO_2) and rough (R-SiO_2) silica nanoparticles (NPs) under glutathione (GSH) condition. (a) Transmission electron microscope (TEM) images of S-SiO_2 NPs. Triplicates were performed independently. (b) Nitrogen adsorption-desorption isotherm of S-SiO_2 NPs along with Barrett-Joyner-Halenda (BJH) pore size distribution plot is shown. (c) TPZ loading content in SiO_2 nanoparticles was measured, n=3 biologically independent samples. (d) TEM images of R-SiO_2–TPZ NPs are shown. Triplicates were performed independently. (e) Hydrodynamic sizes of S-SiO_2 NPs, S-SiO_2–TPZ NPs, R-SiO_2 NPs, and R-SiO_2–TPZ NPs were measured, n=3 biologically independent samples. (f) Hydrodynamic sizes of R-SiO_2–TPZ NPs in the presence of 10 mM GSH were measured at indicated time points, n=3 biologically independent samples. (g) TEM image of R-SiO_2–TPZ NPs incubated with 10 mM GSH for 50 hours is shown. Triplicates were performed independently. (h) Viability of indicated cells after the treatment of debris of R-SiO_2 nanoparticles *in vitro* was measured, n=5 biologically independent samples. (i) *In vitro* viability of indicated neutrophils at 24, 48 and 72 h was measured, n=5 biologically independent samples. All data in this figure are represented as mean ± SD. Source data are provided in the Source data files.

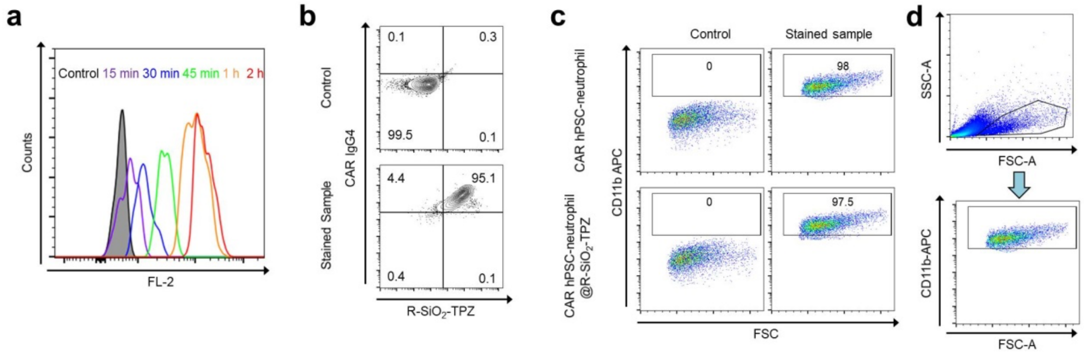

Supplementary Figure 7. **Flow cytometry analysis of CAR-neutrophils after loading TPZ containing rough (R-SiO2) silica nanoparticles (R-SiO2-TPZ NPs).** (a) Flow cytometry analysis of cellular content of R-SiO_2-TPZ NPs in CAR-neutrophils at the indicated time points. (b) Flow cytometry analysis of CAR (IgG4) expression and cellular content of R-SiO_2-TPZ in CAR-neutrophils after loading R-SiO2-TPZ NPs. (c) Flow cytometry analysis of CD11b expression on CAR neutrophils before and after loading R-SiO_2-TPZ NPs. (d) FACS sequential gating strategies for FACS experiments in Supplementary Figure 7c and Supplementary Figure 9f. These results were representative of three biologically independent experiments.

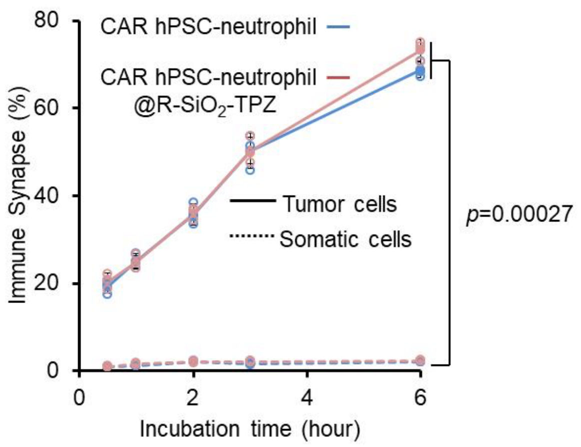

Supplementary Figure 8. **The numbers of immunological synapses formed between indicated neutrophils and tumor cells/normal somatic cells (SVG p12) were measured and quantified,** Data are represented as mean ±SD, one-way analysis of variance (ANOVA). n=3 biologically independent samples. Source data are provided in the Source data files.

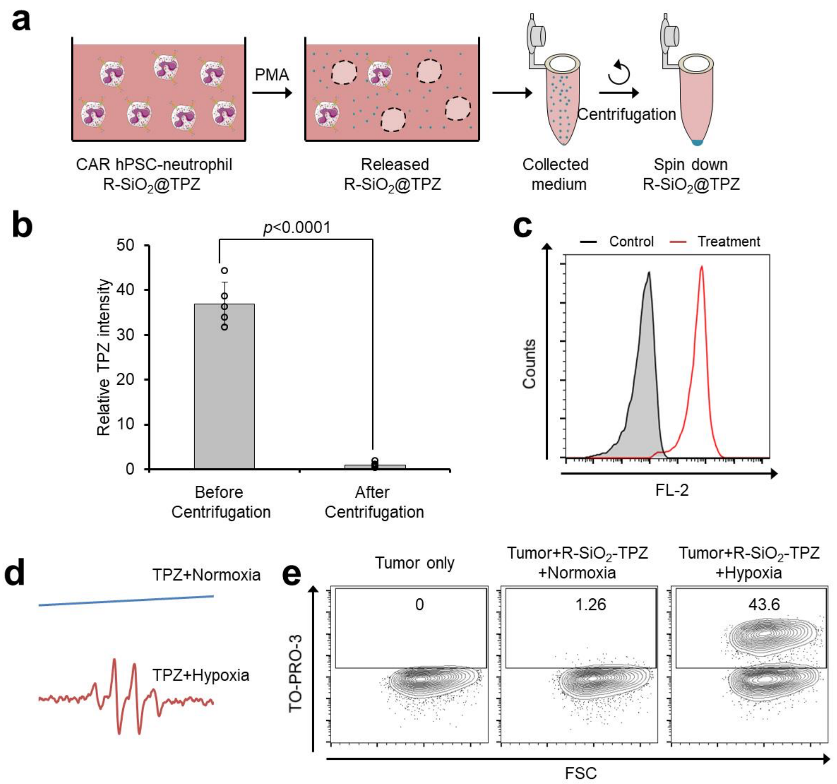

Supplementary Figure 9. The stability and function of R-SiO_2-TPZ NPs within CAR-neutrophils was measured. (a) Schematic of experimental design to investigate the stability of R-SiO_2-TPZ NPS within CAR neutrophils after loading. (b) Relative TPZ intensity before and after centrifugation was measured. n=5 biologically independent samples. Data are represented as mean ± SD, two-tailed Student's *t*-test, *$p<0.05$. (c) Flow cytometry analysis of cellular content of R-SiO_2-TPZ NPs within tumor cells after coculturing with CAR-neutrophils@ R-SiO_2-TPZ NPs was performed. Biological triplicates were performed independently. (d) Electron paramagnetic resonance (EPR) spectroscopy analysis was performed to determine the radicals generation from TPZ under hypoxia and normoxia. (e) Viability of U87MG tumor cells treated by pro-drug TPZ under normoxia and hypoxia was measured by flow cytometry analysis of TO-PRO-3. Biological triplicates were performed independently. Source data are provided in the Source data files.

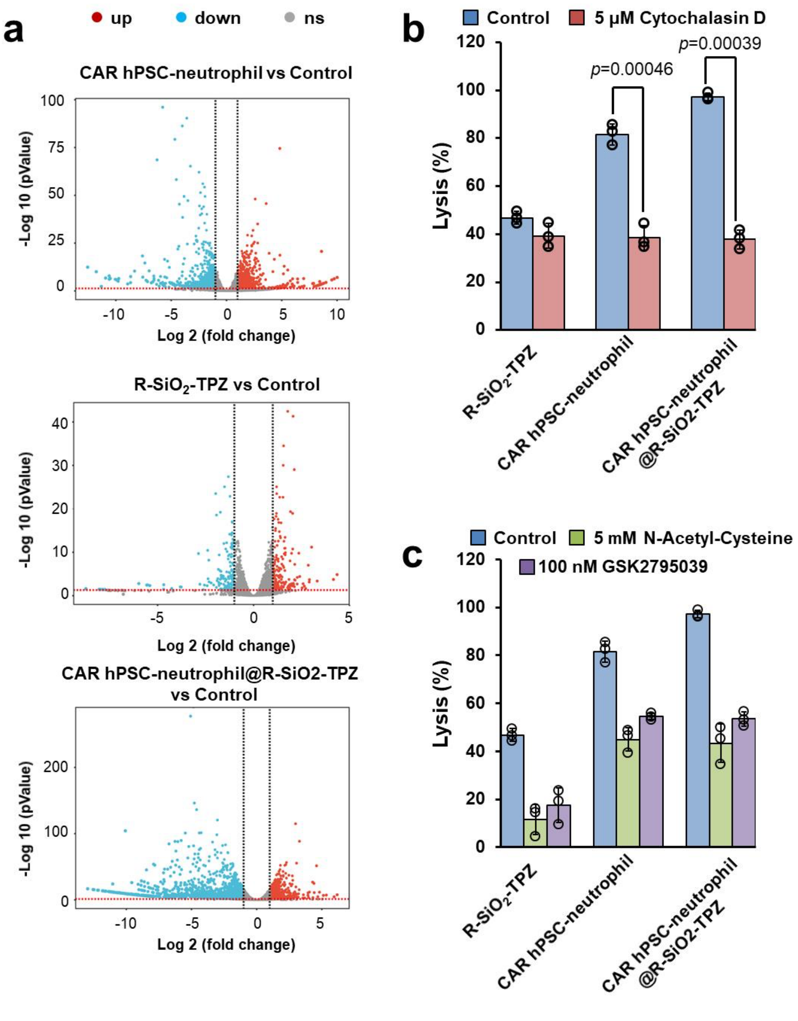

Supplementary Figure 10. **Molecular mechanism investigation of neutrophil-mediated tumor cell lysis under different conditions**. (a) Volcano map of bulk RNA-Seq analysis of U87MG cells under indicated treatments. Tumor lysis of indicated groups treated with 5 μM cytochalasin D (CytoD) (b), 5 mM N-acetyl-cysteine (NAC), or 100 nM GSK2795039 (c) was measured, n=3 biologically independent samples. All data in this figure are represented as mean ± SD, two-tailed Student's *t*-test for (b), *$p<0.05$. Source data are provided in the Source data files.

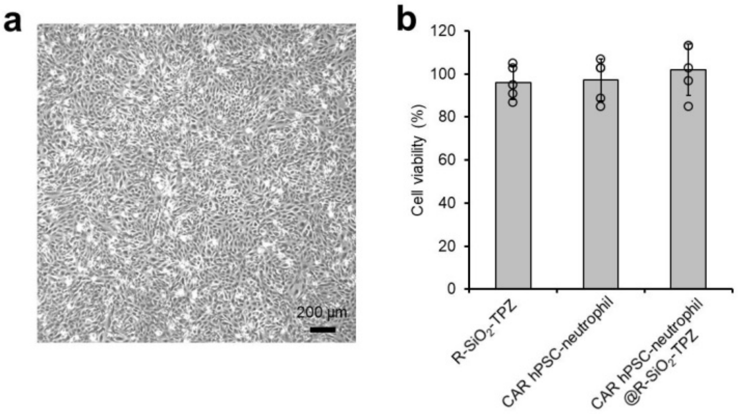

Supplementary Figure 11. **Phenotype and cell viability of endothelial cells in the implemented *in vitro* blood-brain-barrier (BBB) model**. (a) Representative brightfield image of endothelial cells in the implemented *in vitro* BBB model was shown. Scale bar, 200 µm. (b) Endothelial cell viability of indicted nanodrugs and CAR-neutrophils across *in vitro* BBB model was measured 24 hours after cell/nanodrug seeding. n=5 biologically independent samples. Data are represented as mean ± SD. Source data are provided in the Source data files.

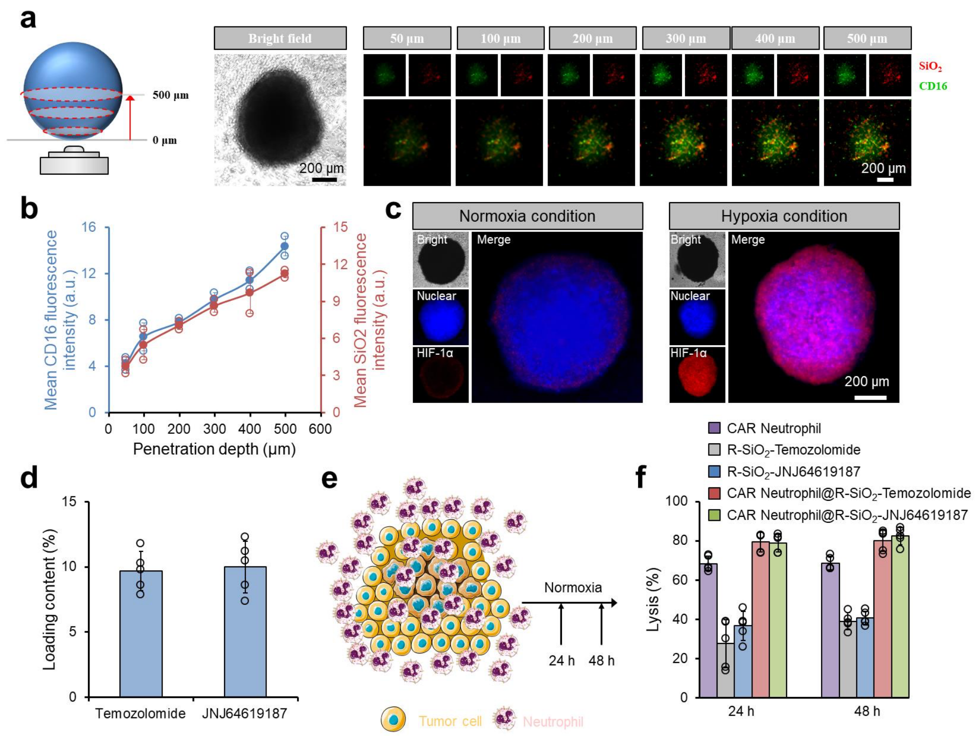

Supplementary Figure 12. **CAR-neutrophil mediated nanodrug delivery for tumor lysis in three-dimensional (3D) model**. (a) Z-section images of SiO_2 and CD16 expression in 3D tumor organoids cocultured with CAR hPSC-neutrophils loaded with R-SiO_2-TPZ. Scale bar: 200 μm. (b) Intensity profiling of SiO_2 and CD16 signals within the 3D tumor organoids, data are represented as mean ± SD of three independent biological replicates. (c) Immunostaining analysis of HIF-1α expression in the 3D tumor under normoxic and hypoxic conditions. Biological triplicates were performed independently. (d) Loading contents of temozolomide (TMZ) and JNJ64619187 in R-SiO_2 nanoparticles were measured. n=5 biologically independent samples. (e) Schematic of neutrophil-infiltrated 3D tumor model is shown. (f) The corresponding tumor-killing ability of indicated neutrophils and nanodrug groups at 24 and 48 hours was measured and quantified using cytotoxicity kit. Data are represented as mean ± SD of five independent replicates. Source data are provided in the Source data files.

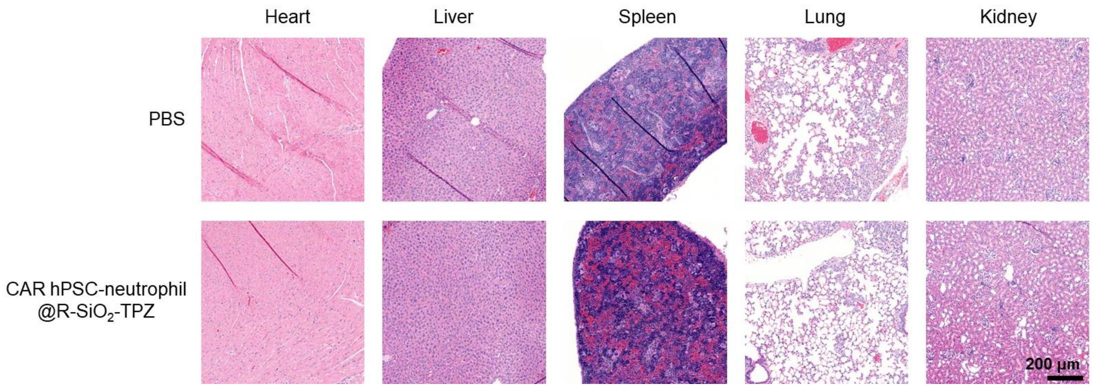

Supplementary Figure 13. H&E images of major organs collected at the end of treatment.

Scale bar, 200 µm. Biological triplicates were performed independently.

.

Supplementary Tables

Supplementary Table 1. Antibodies used in this study

Antibody	Source	Cat. Number	Clone	Lot	Dilution
PE Mouse monoclonal anti human CD45	BD Biosciences	555483	HI30	1264455	1:500
APC Mouse monoclonal anti human CD45	BD Biosciences	555485	HI30	8338537	1:500
FITC Mouse monoclonal anti human CD45	Biolegend	304006	HI30	B233140	1:100
FITC Mouse monoclonal anti human CD34	Miltenyi Biotec	130113178	AC136	5200210484	1:50
APC Mouse monoclonal anti human CD34	Miltenyi Biotec	130113176	AC136	5200809246	1:50
APC Goat polyclonal anti human SOX17	R&D systems	IC1924A	Q9H6I2	ABCF0320081	1:100
Mouse monoclonal anti human VE-cadherin	Miltenyi Biotec	130100742	REA199	5190508238	1:100
Mouse monoclonal anti human CD66b	BD Biosciences	561650	G10F5	9192740	1:100
Mouse monoclonal anti human MPO	Invitrogen	11129941	MPO455-8E6	2243483	1:100
APC Mouse monoclonal anti human CD11b	BD Biosciences	561015	ICRF44	1243950	1:100
FITC Mouse monoclonal anti human CD16	BD Biosciences	556618	3G8	8197598	1:100
FITC Mouse monoclonal anti human CD18	Miltenyi Biotec	130120322	TS1/18	5200706915	1:100
Alex fluor 488 Mouse monoclonal anti human CD14	BD Biosciences	561706	M5E2	2251866	1:100
PE Mouse monoclonal anti human CD15	BD Biosciences	562371	W6D3	B233353	1:100
PE Mouse monoclonal anti human CD44	BD Biosciences	555478	G44-26	0030947	1:100
Acti-Stain 488 Phalloidin	Cytoskeleton, Inc.	PHDG1	n/a	037	1:100
Mouse monoclonal anti human SSEA-4	R&D systems	MAB1435	MC-813-70	JTW5461201	1:100
Goat monoclonal anti human OCT3/4	R&D systems	AF1759	Q01860	JTW0418102	1:100
Goat monoclonal anti ratbit IgG	Cell Signaling	7074S	n/a	28	1:1000
β-Actin (13E5) Rabbit mAb (HRP Conjugate)	Cell Signaling	5125S	13E5	6	1:1000
FITC Mouse Anti-Human IgG4 pFc'	SouthernBiotech	9190-02	HP6023	L3820-M831C	1:1000
Rabbit monoclonal anti human p44/42 MAPK (Erk1/2)	Cell Signaling	4695T	137F5	28	1:1000

Rabbit monoclonal anti human Phospho-p44/42 MAPK (Erk1/2)	Cell Signaling	4370T	D13.14.4E	28	1:1000
Rabbit monoclonal anti human Syk	Cell Signaling	13198T	D3Z1E	9	1:1000
Rabbit monoclonal anti human Phospho-Syk	Cell Signaling	2710T	C87C1	23	1:1000

Supplementary Table 2. Primers used in this study

Genes	Sequences (5' - 3')	Size (bp)/Tm (°C)/Cycles
HIF1α	**F:** GGACAAGTCACCACAGGACA	200/60/35
	**R:** TCAACCGGTTTAAGGACACA	
CLTX-IgG4	**F:** GTTACCACACCCAGCATTCC	220/60/35
	**R:** GGGGGAACAGGAAGACTGAT	
AAVS1 Positive genotyping	**F:** CTGTTTCCCCTTCCCAGGCAGGTCC	991/65/30
	**R:** TCGTCGCGGGTGGCGAGGCGCACCG	
AAVS1 Homozygous screening	**F:** CGGTTAATGTGGCTCTGGTT	204/60/35
	**R:** GAGAGAGATGGCTCCAGGAA	
GAPDH	**F:** CCCCTTCATTGACCTCAACTACA	150/58/30
	**R:** TTGCTGATGATCTTGAGGCTGT	

Source Data-Supplementary Figure 1e

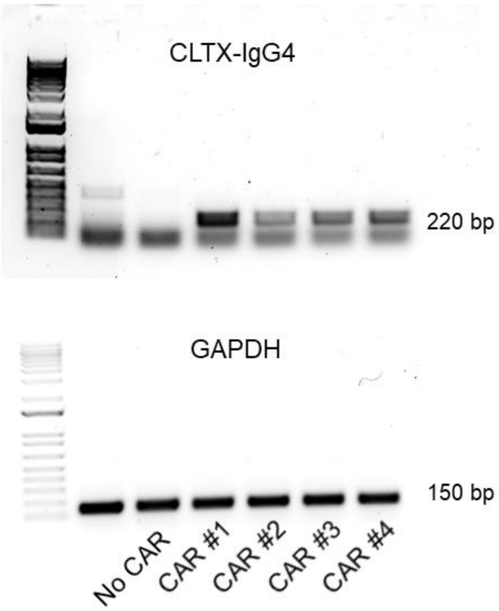

Source Data-Supplementary Figure 5a

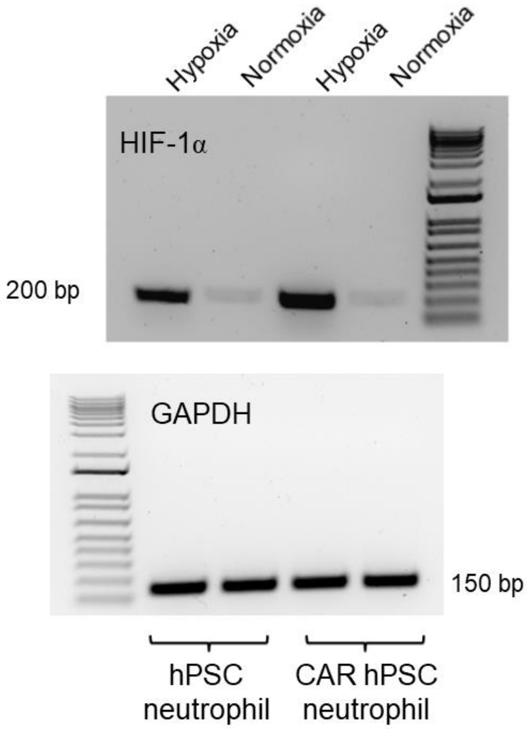

Source Data-Supplementary Figure 5c

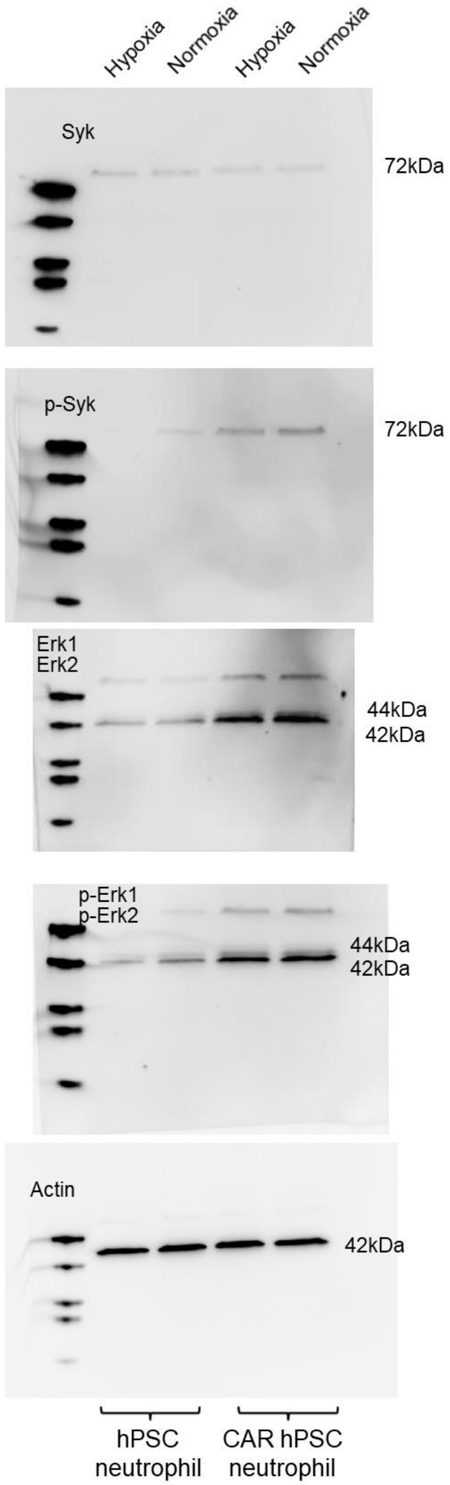

Supplement: Supplementary file 1 — Supplementary Information [file 41467_2023_37872_MOESM1_ESM.pdf]
