# Supplementary material for: CAR-neutrophil mediated delivery of tumor-microenvironment responsive nanodrugs for glioblastoma chemo-immunotherapy
Source: Nat Commun. 2023 Apr 20;14:2266. doi: 10.1038/s41467-023-37872-4 (PMC10119091; doi:10.1038/s41467-023-37872-4)
Supplement: Supplementary file 3 — Reporting Summary [file 41467_2023_37872_MOESM3_ESM.pdf]

nature portfolio

Corresponding author(s): Xiaoping Bao

Last updated by author(s): Mar 18, 2023

Reporting Summary

Nature Portfolio wishes to improve the reproducibility of the work that we publish. This form provides structure for consistency and transparency in reporting. For further information on Nature Portfolio policies, see our Editorial Policies and the Editorial Policy Checklist.

Statistics

For all statistical analyses, confirm that the following items are present in the figure legend, table legend, main text, or Methods section.

n/a	Confirmed	
[ ]	[x]	The exact sample size (*n*) for each experimental group/condition, given as a discrete number and unit of measurement
[ ]	[x]	A statement on whether measurements were taken from distinct samples or whether the same sample was measured repeatedly
[ ]	[x]	The statistical test(s) used AND whether they are one- or two-sided<br>*Only common tests should be described solely by name; describe more complex techniques in the Methods section.*
[x]	[ ]	A description of all covariates tested
[ ]	[x]	A description of any assumptions or corrections, such as tests of normality and adjustment for multiple comparisons
[ ]	[x]	A full description of the statistical parameters including central tendency (e.g. means) or other basic estimates (e.g. regression coefficient) AND variation (e.g. standard deviation) or associated estimates of uncertainty (e.g. confidence intervals)
[ ]	[x]	For null hypothesis testing, the test statistic (e.g. *F*, *t*, *r*) with confidence intervals, effect sizes, degrees of freedom and *P* value noted<br>*Give P values as exact values whenever suitable.*
[x]	[ ]	For Bayesian analysis, information on the choice of priors and Markov chain Monte Carlo settings
[x]	[ ]	For hierarchical and complex designs, identification of the appropriate level for tests and full reporting of outcomes
[x]	[ ]	Estimates of effect sizes (e.g. Cohen's *d*, Pearson's *r*), indicating how they were calculated

Our web collection on statistics for biologists contains articles on many of the points above.

Software and code

Policy information about availability of computer code

Data collection	Leica DMi8 (Leica Microsystems), BD Accuri C6 Plus Flow Cytometry (BD), Illumina NovaSeq 6000 (Illumina),
Data analysis	FlowJo V.10 (FlowJo LLC), Fiji software (ImageJ, v1.51), Excel (Microsoft), HISAT2, rpkmforgenes.py, Morpheus (Broad Institute).

For manuscripts utilizing custom algorithms or software that are central to the research but not yet described in published literature, software must be made available to editors and reviewers. We strongly encourage code deposition in a community repository (e.g. GitHub). See the Nature Portfolio guidelines for submitting code & software for further information.

Data

Policy information about availability of data

All manuscripts must include a data availability statement. This statement should provide the following information, where applicable:

- Accession codes, unique identifiers, or web links for publicly available datasets
- A description of any restrictions on data availability
- For clinical datasets or third party data, please ensure that the statement adheres to our policy

RNA-seq data is accessible via GSE206170: https://www.ncbi.nlm.nih.gov/geo/query/acc.cgi?acc=GSE206170. All the data of this study are available within the article, the Supplementary information file, the Source data file, as described in the Reporting summary of this article. Source data are provided with this study.

Human research participants

Policy information about studies involving human research participants and Sex and Gender in Research.

Reporting on sex and gender	not applicable
Population characteristics	not applicable
Recruitment	not applicable
Ethics oversight	not applicable

Note that full information on the approval of the study protocol must also be provided in the manuscript.

Field-specific reporting

Please select the one below that is the best fit for your research. If you are not sure, read the appropriate sections before making your selection.

[x] Life sciences [ ] Behavioural & social sciences [ ] Ecological, evolutionary & environmental sciences

For a reference copy of the document with all sections, see nature.com/documents/nr-reporting-summary-flat.pdf

Life sciences study design

All studies must disclose on these points even when the disclosure is negative.

Sample size	The number of samples for each assay was indicated in each figure legend. For in vitro cellular assays, the sample sizes (at least three biological replicates) were chosen with these assays yielding statistically significant difference between experimental positive and negative controls and on similar sample sizes. Results are representatives of at least three biological replicates. For in vivo assays, at least 5 mice were chosen for each condition, this sample size was determined by using power calculation for a t-test difference between two independent means based on a normally distributed population with equal variance.
Data exclusions	No relevant data were excluded. A priori criteria for exclusion were developed.
Replication	All experiments were conducted in duplicate or triplicate and reproduced as indicated in the figure legend.
Randomization	For in vivo studies, animals were assigned randomly to experimental and control groups. The rest of the experiments were not randomized, but independent replicates were often performed in different formats and by different investigators, as mitigation measures to cancel out experimental bias. For in vitro studies, samples were also randomly divided into different experimental groups.
Blinding	Blinding was not possible for any experiment as treatment conditions were evident from the data.

Behavioural & social sciences study design

All studies must disclose on these points even when the disclosure is negative.

Study description	*Briefly describe the study type including whether data are quantitative, qualitative, or mixed-methods (e.g. qualitative cross-sectional, quantitative experimental, mixed-methods case study).*
Research sample	*State the research sample (e.g. Harvard university undergraduates, villagers in rural India) and provide relevant demographic information (e.g. age, sex) and indicate whether the sample is representative. Provide a rationale for the study sample chosen. For studies involving existing datasets, please describe the dataset and source.*
Sampling strategy	*Describe the sampling procedure (e.g. random, snowball, stratified, convenience). Describe the statistical methods that were used to predetermine sample size OR if no sample-size calculation was performed, describe how sample sizes were chosen and provide a rationale for why these sample sizes are sufficient. For qualitative data, please indicate whether data saturation was considered, and what criteria were used to decide that no further sampling was needed.*
Data collection	*Provide details about the data collection procedure, including the instruments or devices used to record the data (e.g. pen and paper, computer, eye tracker, video or audio equipment) whether anyone was present besides the participant(s) and the researcher, and whether the researcher was blind to experimental condition and/or the study hypothesis during data collection.*
Timing	*Indicate the start and stop dates of data collection. If there is a gap between collection periods, state the dates for each sample cohort.*

Data exclusions	*If no data were excluded from the analyses, state so OR if data were excluded, provide the exact number of exclusions and the rationale behind them, indicating whether exclusion criteria were pre-established.*
Non-participation	*State how many participants dropped out/declined participation and the reason(s) given OR provide response rate OR state that no participants dropped out/declined participation.*
Randomization	*If participants were not allocated into experimental groups, state so OR describe how participants were allocated to groups, and if allocation was not random, describe how covariates were controlled.*

Ecological, evolutionary & environmental sciences study design

All studies must disclose on these points even when the disclosure is negative.

Study description	*Briefly describe the study. For quantitative data include treatment factors and interactions, design structure (e.g. factorial, nested, hierarchical), nature and number of experimental units and replicates.*
Research sample	*Describe the research sample (e.g. a group of tagged Passer domesticus, all Stenocereus thurberi within Organ Pipe Cactus National Monument), and provide a rationale for the sample choice. When relevant, describe the organism taxa, source, sex, age range and any manipulations. State what population the sample is meant to represent when applicable. For studies involving existing datasets, describe the data and its source.*
Sampling strategy	*Note the sampling procedure. Describe the statistical methods that were used to predetermine sample size OR if no sample-size calculation was performed, describe how sample sizes were chosen and provide a rationale for why these sample sizes are sufficient.*
Data collection	*Describe the data collection procedure, including who recorded the data and how.*
Timing and spatial scale	*Indicate the start and stop dates of data collection, noting the frequency and periodicity of sampling and providing a rationale for these choices. If there is a gap between collection periods, state the dates for each sample cohort. Specify the spatial scale from which the data are taken*
Data exclusions	*If no data were excluded from the analyses, state so OR if data were excluded, describe the exclusions and the rationale behind them, indicating whether exclusion criteria were pre-established.*
Reproducibility	*Describe the measures taken to verify the reproducibility of experimental findings. For each experiment, note whether any attempts to repeat the experiment failed OR state that all attempts to repeat the experiment were successful.*
Randomization	*Describe how samples/organisms/participants were allocated into groups. If allocation was not random, describe how covariates were controlled. If this is not relevant to your study, explain why.*
Blinding	*Describe the extent of blinding used during data acquisition and analysis. If blinding was not possible, describe why OR explain why blinding was not relevant to your study.*

Did the study involve field work? ☐ Yes ☐ No

Field work, collection and transport

Field conditions	*Describe the study conditions for field work, providing relevant parameters (e.g. temperature, rainfall).*
Location	*State the location of the sampling or experiment, providing relevant parameters (e.g. latitude and longitude, elevation, water depth).*
Access & import/export	*Describe the efforts you have made to access habitats and to collect and import/export your samples in a responsible manner and in compliance with local, national and international laws, noting any permits that were obtained (give the name of the issuing authority, the date of issue, and any identifying information).*
Disturbance	*Describe any disturbance caused by the study and how it was minimized.*

Reporting for specific materials, systems and methods

We require information from authors about some types of materials, experimental systems and methods used in many studies. Here, indicate whether each material, system or method listed is relevant to your study. If you are not sure if a list item applies to your research, read the appropriate section before selecting a response.

Materials & experimental systems

n/a | Involved in the study

- [ ] [x] Antibodies
- [ ] [x] Eukaryotic cell lines
- [x] [ ] Palaeontology and archaeology
- [ ] [x] Animals and other organisms
- [x] [ ] Clinical data
- [x] [ ] Dual use research of concern

Methods

n/a | Involved in the study

- [ ] [x] ChIP-seq
- [ ] [x] Flow cytometry
- [x] [ ] MRI-based neuroimaging

Antibodies

Antibodies used

Antibody Source Cat. Number Clone Lot Dilution
PE Mouse monoclonal anti human CD45 BD Biosciences 555483 HI30 1264455 1:500
APC Mouse monoclonal anti human CD45 BD Biosciences 555485 HI30 8338537 1:500
FITC Mouse monoclonal anti human CD45 Biolegend 304006 HI30 B233140 1:100
FITC Mouse monoclonal anti human CD34 Miltenyi Biotec 130113178 AC136 5200210484 1:50
APC Mouse monoclonal anti human CD34 Miltenyi Biotec 130113176 AC136 5200809246 1:50
APC Goat polyclonal anti human SOX17 R&D systems IC1924A Q9H6I2 ABCF0320081 1:100
Mouse monoclonal anti human VE-cadherin Miltenyi Biotec 130100742 REA199 5190508238 1:100
Mouse monoclonal anti human CD66b BD Biosciences 561650 G10F5 9192740 1:100
Mouse monoclonal anti human MPO Invitrogen 11129941 MPO455-8E6 2243483 1:100
APC Mouse monoclonal anti human CD11b BD Biosciences 561015 ICRF44 1243950 1:100
FITC Mouse monoclonal anti human CD16 BD Biosciences 556618 3G8 8197598 1:100
FITC Mouse monoclonal anti human CD18 Miltenyi Biotec 130120322 TS1/18 5200706915 1:100
Alex fluor 488 Mouse monoclonal anti human CD14 BD Biosciences 561706 M5E2 2251866 1:100
PE Mouse monoclonal anti human CD15 BD Biosciences 562371 W6D3 B233353 1:100
PE Mouse monoclonal anti human CD44 BD Biosciences 555478 G44-26 0030947 1:100
Acti-Stain 488 Phalloidin Cytoskeleton, Inc. PHDG1 n/a 037 1:100
Mouse monoclonal anti human SSEA-4 R&D systems MAB1435 MC-813-70 JTW5461201 1:100
Goat monoclonal anti human OCT3/4 R&D systems AF1759 Q01860 JTW0418102 1:100
Goat monoclonal anti ratbit IgG Cell Signaling 7074S n/a 28 1:1000
β-Actin (13E5) Rabbit mAb (HRP Conjugate) Cell Signaling 5125S 13E5 6 1:1000
FITC Mouse Anti-Human IgG4 pFc' SouthernBiotech 9190-02 HP6023 L3820-M831C 1:1000
Rabbit monoclonal anti human p44/42 MAPK (Erk1/2) Cell Signaling 4695T 137F5 28 1:1000
Rabbit monoclonal anti human Phospho-p44/42 MAPK (Erk1/2) Cell Signaling 4370T D13.14.4E 28 1:1000
Rabbit monoclonal anti human Syk Cell Signaling 13198T D3Z1E 9 1:1000
Rabbit monoclonal anti human Phospho-Syk Cell Signaling 2710T C87C1 23 1:1000

Validation

Antibodies were validated using positive and negative cells or isotype controls. Validation reports were provided by the antibody manufacturers. Appropriate compensation controls were used for every experiment. The BD Accuri C6 plus was calibrated daily using CS&T beads (BD Biosciences) or manufacturer recommended methods.
The manufacturers's instruction below:
Human CD45 (Biosciences, Cat:555483)
https://www.bdbiosciences.com/en-nz/products/reagents/flow-cytometry-reagents/research-reagents/single-color-antibodies-ruo/pe-mouse-anti-human-cd45.561866
Human CD45 (Biosciences, Cat:555485)
https://www.bdbiosciences.com/en-us/products/reagents/flow-cytometry-reagents/research-reagents/single-color-antibodies-ruo/apc-mouse-anti-human-cd45.555485
Human CD45 (Biolegend, Cat:304006)
https://www.biolegend.com/fr-ch/products/fitc-anti-human-cd45-antibody-707
Human CD34 (Miltenyi Biotec, Cat:130113178)
https://www.miltenyibiotec.com/US-en/products/cd34-antibody-anti-human-ac136.html#fitc:100-tests-in-200-ul
Human SOX17 (R&D systems, Cat: IC1924A)
https://www.rndsystems.com/products/human-sox17-apc-conjugated-antibody_ic1924a
Human VE-cadherin (Santa Cruz, Cat: sc-9989)
https://www.scbt.com/p/ve-cadherin-antibody-f-8
Human CD66b (Biosciences, Cat:561650)
https://www.bdbiosciences.com/en-us/search-results?searchKey=561650
Human MPO (Invitrogen, Cat: 11129941)
https://www.thermofisher.com/antibody/product/Myeloperoxidase-MPO-Antibody-clone-MPO455-8E6-Monoclonal/11-1299-41
Human CD11b (Biosciences, Cat:561015)
https://www.bdbiosciences.com/en-us/products/reagents/flow-cytometry-reagents/research-reagents/single-color-antibodies-ruo/apc-mouse-anti-human-cd11b.561015

Human CD16 (Biosciences, Cat:556618)
https://www.bdbiosciences.com/en-us/products/reagents/flow-cytometry-reagents/research-reagents/single-color-antibodies-ruo/fitc-mouse-anti-human-cd16.556618
Human CD18 (Miltenyi Biotec, Cat:130120322)
https://www.miltenyibiotec.com/US-en/products/cd18-antibody-anti-human-ts1-18.html#biotin:100-tests-in-1-ml
Human CD14 (Biosciences, Cat:561706)
https://www.bdbiosciences.com/en-us/products/reagents/flow-cytometry-reagents/research-reagents/single-color-antibodies-ruo/alexa-fluor-488-mouse-anti-human-cd14.561706
Human CD15 (Biosciences, Cat:562371)
https://www.bdbiosciences.com/en-us/products/reagents/flow-cytometry-reagents/research-reagents/single-color-antibodies-ruo/pe-mouse-anti-human-cd15.562371
Human CD44 (Biosciences, Cat:555478)
https://www.bdbiosciences.com/en-us/products/reagents/flow-cytometry-reagents/research-reagents/single-color-antibodies-ruo/fitc-mouse-anti-human-cd44.555478
Acti-Stain 488 Phalloidin
https://www.cytoskeleton.com/actin/acti-stain/phdg1
Human SSEA-4 (Santa Cruz, Cat: sc-21704)
https://www.scbt.com/p/ssea-4-antibody-813-70?requestFrom=search
Human OCT3/4 (Santa Cruz, Cat: sc-5279)
https://www.scbt.com/p/oct-3-4-antibody-c-10?requestFrom=search
Goat monoclonal anti ratbit IgG (Cell Signaling, Cat: 7074S)
https://www.cellsignal.com/products/secondary-antibodies/anti-rabbit-igg-hrp-linked-antibody/7074?_=1678990930444&Ntt=7074&tahead=true
β-Actin (13E5) Rabbit mAb (Cell Signaling, Cat: 5125S)
https://www.cellsignal.com/products/antibody-conjugates/b-actin-13e5-rabbit-mab-hrp-conjugate/5125?site-search-type=Products&N=4294956287&Ntt=5125s&fromPage=plp&_requestid=582512
Human IgG4 pFc (SouthernBiotech, Cat: 9190-02)
https://www.southernbiotech.com/mouse-anti-human-igg4-pfc-fitc-hp6023-9190-02
Human p44/42 MAPK (Erk1/2) (Cell Signaling, Cat: 4695T)
https://www.cellsignal.com/products/primary-antibodies/p44-42-mapk-erk1-2-137f5-rabbit-mab/4695?site-search-type=Products&N=4294956287&Ntt=4695t&fromPage=plp&_requestid=594955
Human Phospho-p44/42 MAPK (Erk1/2) (Cell Signaling, Cat: 4370T)
https://www.cellsignal.com/products/primary-antibodies/phospho-p44-42-mapk-erk1-2-thr202-tyr204-d13-14-4e-xp-rabbit-mab/4370?site-search-type=Products&N=4294956287&Ntt=4370t&fromPage=plp&_requestid=595024
Human Syk (Cell Signaling, Cat: 13198T)
https://www.cellsignal.com/products/primary-antibodies/syk-d3z1e-xp-rabbit-mab/13198?site-search-type=Products&N=4294956287&Ntt=13198t&fromPage=plp&_requestid=595190
Human Phospho-Syk (Cell Signaling, Cat: 2710T)
https://www.cellsignal.com/products/primary-antibodies/phospho-syk-tyr525-526-c87c1-rabbit-mab/2710?site-search-type=Products&N=4294956287&Ntt=2710t&fromPage=plp&_requestid=595291

Eukaryotic cell lines

Policy information about cell lines and Sex and Gender in Research

Cell line source(s)	H9 cells were obtained from WiCell, HBEC-5i cells were obtained from ATCC,and U87MG cells were acquired from Dr. Sandro AT Purdue University.
Authentication	Cell lines were authenticated as by routine practice by WiCell. Cell lines cultured in-house were validated via karyotyping by WiCell services. Morphology and relevant antigen expression was routinely validated during culture.
Mycoplasma contamination	Cell lines were tested for the presence of mycoplasma contamination (MycoAlert™ Mycoplasma Detection Kit, LT07-318, Lonza, Basel, Switzerland). All the cell line were negative for mycoplsma.
Commonly misidentified lines (See ICLAC register)	None of the cell lines used in this manuscript are listed in the ICLAC Database of Cross-contaminated or Misidentified Cell Lines (Version 8.0).

Palaeontology and Archaeology

Specimen provenance	*Provide provenance information for specimens and describe permits that were obtained for the work (including the name of the issuing authority, the date of issue, and any identifying information). Permits should encompass collection and, where applicable, export.*
Specimen deposition	*Indicate where the specimens have been deposited to permit free access by other researchers.*

Dating methods	*If new dates are provided, describe how they were obtained (e.g. collection, storage, sample pretreatment and measurement), where they were obtained (i.e. lab name), the calibration program and the protocol for quality assurance OR state that no new dates are provided.*

☐ Tick this box to confirm that the raw and calibrated dates are available in the paper or in Supplementary Information.

Ethics oversight	*Identify the organization(s) that approved or provided guidance on the study protocol, OR state that no ethical approval or guidance was required and explain why not.*

Note that full information on the approval of the study protocol must also be provided in the manuscript.

Animals and other research organisms

Policy information about studies involving animals; ARRIVE guidelines recommended for reporting animal research, and Sex and Gender in Research

Laboratory animals	Immunodeficient mice: NOD.Cg-RAG1tm1MomIL2rgtm1Wjl/SzJ (NRG), all the female mice used in this study were 6- to 10-week-old. Mice were housed in pathogen free and ventilated cages, and allowed free access to auto claved food and water, in a 12 hour light/dark cycle, with room temperature at 21±2 degree and humidity between 45 and 65%.
Wild animals	This study did not involve wild animals.
Reporting on sex	Female 6- to 10-week old immunodeficient NOD.Cg-RAG1tm1MomIL2rgtm1Wjl/SzJ (NRG) mice were used in this study.
Field-collected samples	No field-collected samples were used in this study.
Ethics oversight	All mouse experiments were approved by the Purdue Animal Care and Use Committee (PACUC).

Note that full information on the approval of the study protocol must also be provided in the manuscript.

Clinical data

Policy information about clinical studies

All manuscripts should comply with the ICMJEguidelines for publication of clinical research and a completedCONSORT checklist must be included with all submissions.

Clinical trial registration	*Provide the trial registration number from ClinicalTrials.gov or an equivalent agency.*
Study protocol	*Note where the full trial protocol can be accessed OR if not available, explain why.*
Data collection	*Describe the settings and locales of data collection, noting the time periods of recruitment and data collection.*
Outcomes	*Describe how you pre-defined primary and secondary outcome measures and how you assessed these measures.*

Dual use research of concern

Policy information about dual use research of concern

Hazards

Could the accidental, deliberate or reckless misuse of agents or technologies generated in the work, or the application of information presented in the manuscript, pose a threat to:

No	Yes	
☐	☐	Public health
☐	☐	National security
☐	☐	Crops and/or livestock
☐	☐	Ecosystems
☐	☐	Any other significant area

Experiments of concern

Does the work involve any of these experiments of concern:

No	Yes	
☐	☐	Demonstrate how to render a vaccine ineffective
☐	☐	Confer resistance to therapeutically useful antibiotics or antiviral agents
☐	☐	Enhance the virulence of a pathogen or render a nonpathogen virulent
☐	☐	Increase transmissibility of a pathogen
☐	☐	Alter the host range of a pathogen
☐	☐	Enable evasion of diagnostic/detection modalities
☐	☐	Enable the weaponization of a biological agent or toxin
☐	☐	Any other potentially harmful combination of experiments and agents

ChIP-seq

Data deposition

☒ Confirm that both raw and final processed data have been deposited in a public database such as GEO.

☐ Confirm that you have deposited or provided access to graph files (e.g. BED files) for the called peaks.

Data access links
May remain private before publication.

https://www.ncbi.nlm.nih.gov/geo/query/acc.cgi?acc=GSE206170

Files in database submission

GSM6245281	U87MG Control1
GSM6245282	U87MG Control2
GSM6245283	GBM CAR neuttrophil_1
GSM6245284	GBM CAR neuttrophil_2
GSM6245285	GBM Nanodrug_1
GSM6245286	GBM Nanodrug_2
GSM6245287	GBMXB CAR neutrophil Nanodrug_1
GSM6245288	GBMXB CAR neutrophil Nanodrug_2

Genome browser session
(e.g. UCSC)

Provide a link to an anonymized genome browser session for "Initial submission" and "Revised version" documents only, to enable peer review. Write "no longer applicable" for "Final submission" documents.

Methodology

Replicates

Describe the experimental replicates, specifying number, type and replicate agreement.

Sequencing depth

Describe the sequencing depth for each experiment, providing the total number of reads, uniquely mapped reads, length of reads and whether they were paired- or single-end.

Antibodies

Describe the antibodies used for the ChIP-seq experiments; as applicable, provide supplier name, catalog number, clone name, and lot number.

Peak calling parameters

Specify the command line program and parameters used for read mapping and peak calling, including the ChIP, control and index files used.

Data quality

Describe the methods used to ensure data quality in full detail, including how many peaks are at FDR 5% and above 5-fold enrichment.

Software

Describe the software used to collect and analyze the ChIP-seq data. For custom code that has been deposited into a community repository, provide accession details.

Flow Cytometry

Plots

Confirm that:

- [x] The axis labels state the marker and fluorochrome used (e.g. CD4-FITC).
- [x] The axis scales are clearly visible. Include numbers along axes only for bottom left plot of group (a 'group' is an analysis of identical markers).
- [x] All plots are contour plots with outliers or pseudocolor plots.
- [x] A numerical value for number of cells or percentage (with statistics) is provided.

Methodology

Sample preparation	Standard flow cytometry protocol was used in this study. Cells were washed before and after staining, and Fc block was used when staining Fc receptor expressing cells. Samples were acquired on an BD Accuri C6 plus. Compensation was performed with every experiment and the instrument was calibrated daily using CS&T beads. Samples preparation details on individual experiments are in the Methods section.
Instrument	Flow cytometry was performed on BD Accuri C6 plus (Becton-Dickinson).
Software	FCS files were analyzed with FlowJo V10.
Cell population abundance	The abundance of the cells in total collected samples is around 20%.
Gating strategy	Generally, FACS gating was performed as follows: FSC/SSC→Singlets→Live cells→gating of interest. The appropriate negative control was used for generating gates of interest. Please see Supplementary Figure 1g, Supplementary Figure 4f, and Supplementary Figure 7d for details.

- [x] Tick this box to confirm that a figure exemplifying the gating strategy is provided in the Supplementary Information.

Magnetic resonance imaging

Experimental design

Design type	*Indicate task or resting state; event-related or block design.*
Design specifications	*Specify the number of blocks, trials or experimental units per session and/or subject, and specify the length of each trial or block (if trials are blocked) and interval between trials.*
Behavioral performance measures	*State number and/or type of variables recorded (e.g. correct button press, response time) and what statistics were used to establish that the subjects were performing the task as expected (e.g. mean, range, and/or standard deviation across subjects).*

Acquisition

Imaging type(s)	*Specify: functional, structural, diffusion, perfusion.*
Field strength	*Specify in Tesla*
Sequence & imaging parameters	*Specify the pulse sequence type (gradient echo, spin echo, etc.), imaging type (EPI, spiral, etc.), field of view, matrix size, slice thickness, orientation and TE/TR/flip angle.*
Area of acquisition	*State whether a whole brain scan was used OR define the area of acquisition, describing how the region was determined.*

Diffusion MRI ☐ Used ☐ Not used

Preprocessing

Preprocessing software	*Provide detail on software version and revision number and on specific parameters (model/functions, brain extraction, segmentation, smoothing kernel size, etc.).*
Normalization	*If data were normalized/standardized, describe the approach(es): specify linear or non-linear and define image types used for transformation OR indicate that data were not normalized and explain rationale for lack of normalization.*
Normalization template	*Describe the template used for normalization/transformation, specifying subject space or group standardized space (e.g. original Talairach, MNI305, ICBM152) OR indicate that the data were not normalized.*

Noise and artifact removal | *Describe your procedure(s) for artifact and structured noise removal, specifying motion parameters, tissue signals and physiological signals (heart rate, respiration).*

Volume censoring | *Define your software and/or method and criteria for volume censoring, and state the extent of such censoring.*

Statistical modeling & inference

Model type and settings | *Specify type (mass univariate, multivariate, RSA, predictive, etc.) and describe essential details of the model at the first and second levels (e.g. fixed, random or mixed effects; drift or auto-correlation).*

Effect(s) tested | *Define precise effect in terms of the task or stimulus conditions instead of psychological concepts and indicate whether ANOVA or factorial designs were used.*

Specify type of analysis: ☐ Whole brain ☐ ROI-based ☐ Both

Statistic type for inference (See Eklund et al. 2016) | *Specify voxel-wise or cluster-wise and report all relevant parameters for cluster-wise methods.*

Correction | *Describe the type of correction and how it is obtained for multiple comparisons (e.g. FWE, FDR, permutation or Monte Carlo).*

Models & analysis

n/a	Involved in the study
☐	☐ Functional and/or effective connectivity
☐	☐ Graph analysis
☐	☐ Multivariate modeling or predictive analysis

Functional and/or effective connectivity | *Report the measures of dependence used and the model details (e.g. Pearson correlation, partial correlation, mutual information).*

Graph analysis | *Report the dependent variable and connectivity measure, specifying weighted graph or binarized graph, subject- or group-level, and the global and/or node summaries used (e.g. clustering coefficient, efficiency, etc.).*

Multivariate modeling and predictive analysis | *Specify independent variables, features extraction and dimension reduction, model, training and evaluation metrics.*
